# Supplementary material for: Postnatal Development of Centrifugal Inputs to the Olfactory Bulb
Source: Front Neurosci. 2022 Feb 24;16:815282. doi: 10.3389/fnins.2022.815282 (PMC8908425; doi:10.3389/fnins.2022.815282)
Supplement: Supplementary file 1 [file Data_Sheet_1.pdf]

Supplementary Material

Postnatal development of centrifugal inputs to the olfactory bulb

Table S1 (Relates to Figure 2). Fitting parameters for the quantification of cell density across development in Figure 2.

$f(x) = p_1 * x + p_2$

	p_1	p_2	R^2
**AON**	-16.03	3096	0.221
**nLOT**	-1.705	2603	0.008
**aPIR**	-7.458	2655	0.200
**iPIR**	-15.22	2649	0.197
**pPIR**	-15.15	2578	0.276
**CoA**	-16.73	2883	0.375
**LEC**	-14.27	2501	0.375
**vCA1**	-17.19	2863	0.462

Table S2 (Relates to Figures 3, 4, 5). Posthoc comparison for ANOVAs. Asterisks indicate significance ($p<0.05$*, $p<0.01$**, $p<0.001$***).

		AON ipsi	AON contra	nLOT ipsi	nLOT contra	aPIR	iPIR	pPIR	CoA	LEC	vCA1
**P0**	**P3**	0.9999	0.9999	0.9876	0.9999	0.5037	1	0.9999	1	1	1
**P0**	**P6**	0.5162	0.8641	0.9127	0.9828	0.5578	0.9151	0.9997	0.9999	0.9999	0.9999
**P0**	**P9**	0.0146 *	0.0794	0.1890	0.4200	0.0274 *	0.4496	0.5539	0.3797	0.1438	0.3568
**P0**	**P12**	0.0005 ***	0.1488	0.0244 *	0.3402	0.0017 **	0.1354	0.5266	0.4928	0.4509	0.8551
**P0**	**P49**	1.31e^{-5} ***	0.0031 **	9.40e^{-5} ***	0.0028 **	0.0001 ***	0.0024 **	0.0004 ***	0.0181 *	0.0001 ***	3.29e^{-5} ***
**P3**	**P6**	0.7265	0.8487	0.9992	0.9784	0.9999	0.9532	0.9989	0.9999	0.9999	0.9999
**P3**	**P9**	0.0644	0.1210	0.6990	0.4997	0.8861	0.6338	0.5998	0.5276	0.2742	0.5045
**P3**	**P12**	0.0034 **	0.1984	0.1945	0.4174	0.2305	0.2610	0.5708	0.6274	0.6164	0.9112
**P3**	**P49**	9.32e^{-5} ***	0.0068 **	0.0013 **	0.0068 **	0.0194 *	0.0083 **	0.0011 **	0.0428 *	0.0008 ***	0.0001 ***
**P6**	**P9**	0.7422	0.7716	0.9005	0.9348	0.8427	0.9945	0.8453	0.6658	0.2201	0.5670
**P6**	**P12**	0.0900	0.8903	0.3584	0.8777	0.1963	0.8035	0.8175	0.7582	0.5338	0.9420
**P6**	**P49**	0.0019 **	0.0910	0.0029 **	0.0339 *	0.0160 *	0.0566	0.0027 **	0.0648	0.0006 ***	0.0001 ***
**P9**	**P12**	0.3056	0.9995	0.7020	0.9997	0.4890	0.8994	0.9999	0.9999	0.9590	0.9054
**P9**	**P49**	0.0022 **	0.2806	0.0016 **	0.0337 *	0.0230 *	0.0252 *	0.0021 **	0.2764	0.0059 **	0.0001 ***
**P12**	**P49**	0.1389	0.2063	0.0314 *	0.0677	0.4602	0.1793	0.0037 **	0.2472	0.0018 **	5.24e^{-5} ***
